# Role of Proton Beam Therapy in Hepatic Oligometastasis: Review of Evidence

**DOI:** 10.15388/Amed.2025.32.1.25

**Published:** 2025-02-18

**Authors:** Satyajeet Rath

**Affiliations:** All India Institute of Medical Sciences, Rajkot, India

**Keywords:** oligometastatic cancers, proton beam therapy, hepatic oligometastasis, liver secondary, toxicity profile, oligometastatiniai vėžiai, protonų spindulių terapija, kepenų oligometastazės, kepenų metastazės, toksiškumo profilis

## Abstract

Hepatic oligometastasis (hOMC) incidence varies from 10-40% in the literature. While the old standard for local treatment was surgical resection, options like TACE, TARE, SBRT with photons and off late protons have come to the fore. The proton beam therapy (PBT) use has gradually started to get adopted in all regions worldwide with increasingly better availability and ever-reducing costs. The role of PBT in hOMC has been studied in many retrospective cohort studies. Although there is a plethora of evidence on photon-SBRT, there are very few analyses on the role of PBT in hOMC. The author intends to analyse the efficacy in terms of the local control (LC) and the overall survival (OS) for PBT and its toxicity profile in this systematic review. LC remains persistently high (76–89% at 1–2 years) across breast, colo-rectum, stomach, and esophagus. Sites like colo-rectum and breast show relatively better survival outcomes, with progression free survival (35–52% at 1–2 years) and OS (33–78% at 1–2 years) than other sites, likely due to disease biology. Breast primary derived hOMCs had the best 3-year OS of 67.6%. Toxicities remain remarkably low with grade 3 plus toxicities ranging from 0–3%, which reflects the ability of proton therapy to deliver beams with precision.

## Abstract (it)

Literatūroje nurodomas kepenų oligometastazių (hOMC) dažnis svyruoja nuo 10 iki 40 procentų. Senasis vietinio gydymo standartas buvo chirurginė rezekcija, o dabar visų pirma vertinamos tokios gydymo galimybės kaip TACE, TARE, SBRT (stereotaksinė kūno spindulinė terapija) su fotonais ir vėlyvaisiais protonais. Protonų spindulių terapija (PBT) pamažu plinta visame pasaulyje, nes jos prieinamumas vis gerėja, o išlaidos nuolat mažėja. PBT veiksmingumas esant hOMC buvo tiriamas daugelyje retrospektyvinių kohortinių tyrimų. Nors yra gausu įrodymų apie fotonų SBRT, labai mažai vertintas PBT veiksmingumas esant hOMC. Sisteminėje apžvalgoje analizuojamas PBT veiksmingumas vietinei kontrolei (LC) ir bendram išgyvenamumui (OS) bei PBT toksiškumo profilis. LC išlieka nuolat aukštas (76–89 % per 1–2 metus) krūties, storosios žarnos, skrandžio ir stemplės vėžio atvejais. Sergant tokių organų kaip storosios žarnos ir krūties vėžiu išgyvenamumo rezultatai santykinai geresni, be progresavimo (35–52 % per 1–2 metus) ir OS (33–78 % per 1–2 metus) nei sergant kitų organų vėžiu, greičiausiai dėl ligos biologijos. Pirminės krūties hOMC turėjo geriausią 3 metų OS – 67,6 %. Toksiškumo poveikis išlieka labai mažas, 3 laipsnio ir didesnio toksinio poveikio dažnis svyruoja nuo 0 iki 3 %, o tai rodo protonų terapijos gebėjimą tiksliai nukreipti spindulius.

## Introduction

Oligometastatic cancers (OMCs) are broadly considered as cancers which fall within the ambit of potentially curable cancers. However, the presence of metastasis itself portends worse prognosis as opposed to a non-metastatic status. OMCs are broadly defined as cancers with limited [1–5] metastatic lesions, all amenable for local ablative therapy. Although the definition varies from one source to another, the presently mentioned definition can be broadly considered to encompass the whole spectrum of OMCs with minor variations [1–2]. The incidence of OMCs varies from less than 1% in lung cancer to around up to 10–40% in liver malignancies [3]. Liver is the second most common site of metastasis from any primary tumour [4,5]. Liver metastatectomy has been the traditional standard for hepatic oligo-metastasis (hOMCs) in tumours like colon cancers [6,7].

OMCs are amenable to local treatment modalities, and proton beam radiotherapy (PBT) is a recent and interesting addition to the armoire of radiation oncologists for the local management of liver OMCs. Around 100 PBT treatment centers are operational in the world at the present date with more than 50 under construction, with the total number of patients being treated amounting to over 0.3 million cases by 2022 [8]. Stereotactic radiotherapy (SRT) has been widely used for treatment of metastasis in OMCs [9]. Proton therapy is a viable option as a locally directed therapy as an alternative to surgical removal of metastases. While photon radio-therapy (pRT) has been widely used for treating OMCs in the last decade [10,11], the use of PBTs has been sparse, mainly because of the limited availability and costs involved.

Although there are several reports about the use of PBT in liver secondaries, concise review about the use of protons, doses, outcomes and toxicity data is lacking. To address the lack of data, the author intends to analyse and present the data of the PBT use in metastasis-directed therapies in a concise manner for liver secondaries from any primary site.

The author intends to analyse the effectiveness of PBT as well as analyse the toxicity rates. The purpose of the review is to establish as to whether PBT can be considered as comparable to pRT in treating hepatic OMCs from any primary site in terms of the outcomes and the safety profile. The secondary end point of the study was to analyse whether liver OMCs from any particular site portend better prognosis as compared to other sites.

## Material and Methods

### Search Strategy

A systematic search of the extant literature was performed. The search intended to identify all published researches evaluating the proton beam therapy for hepatic oligometastasis. The databases searched included *PubMed, Embase, Scopus*, and *Google Scholar*. The following terms were used for search: ‘protons in liver oligometastasis’, ‘protons in hepatic oligometastasis’, ‘protons in liver metastasis’ and ‘protons in hepatic metastasis’. The search was further broadened by extensive cross-evaluation of all the refences in the relevant articles fulfilling the earmarked inclusion criteria in order to identify eventual additional non-indexed literature.

### Eligibility Criteria

All the studies which treated oligometastatic liver disease derived from any primary site with PBT were included in the final analysis. Studies written in any language other than English were not considered for the final evaluation. Studies in which poly-metastatic disease was treated with PBT were excluded from the final review. Dosimetric studies were not included in the final evaluation.

### Definition

Although a consistent and precise definition of oligometastatic disease is lacking, for the purposes of consideration of this critical review, all metastatic disease with 1–5 metastatic sites in total, including liver, were considered as OMCs. Broadly speaking, oligometastatic disease is defined as a favourable clinical entity, with limited metastatic sites, with potential for possible locally ablative treatment [12,13]. Liver OMCs with a primary tumour from any site were taken into consideration.

**Figure 1:**
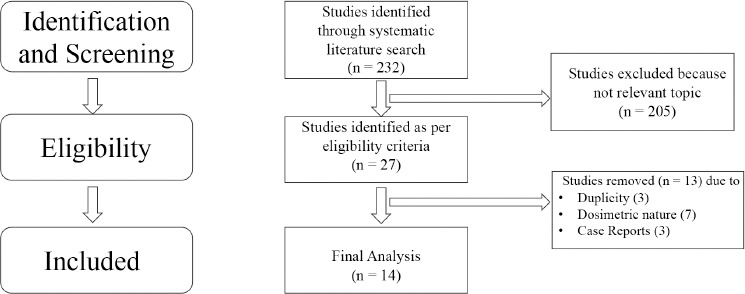
Diagram with selection of the studies for review

A total of 232 articles were identified after the primary literature search. After abstract and title review, a total of twenty-seven eligible articles/abstracts were considered for the full text review. Following the final review of all the publications, 14 articles were considered for the final review. Three articles were removed due to data duplicity, three were case reports, and seven studies were of non-clinical, dosimetric nature. Of the final fourteen studies considered for review, two were abstracts and the twelve texts were full papers (Figure 1). Data collection included key details like the year of publication, the abstract/full paper, authorship, the study type – i.e., prospective/retrospective, case report/series/full paper, the number of patients included in the study/report, survival outcomes and toxicities. All the publications, except two, were of retrospective nature. Statistical analysis was performed by using *SPSS*, a version issued in 2023. P <0.05 was considered as significant.

## Discussion

Liver metastasis represents the most frequent type of liver cancer [14]. Systemic chemotherapy is the primary treatment of choice in hepatic metastasis [15,16]. Various local therapy options like surgery for the removal of metastasis, radiofrequency ablation, trans-arterial chemo embolization (TACE), trans-arterial radio-embolization (TARE) and radiotherapy are used for effective local control [17].

Radiotherapy (RT) is an excellent local treatment option, and specifically liver stereotactic body radiotherapy (SBRT) gives excellent local control rates of approximately 80% [18,19], although it slightly increases the inherent risk of radiation-induced liver disease (RILD) [20,21]. The modern conformal RT delivery techniques using PBT allow us to deliver high-dose radiation therapy to a small volume of liver without any gross adverse effects related to large-volume irradiation of normal healthy liver [22].

The rationale for using PBT in hOMC lies in its ability to deliver high-dose RT to the tumour while controlling doses to the surrounding normal tissues, but technical and clinical challenges like motion management, determining the adequate margin, the proper dose fractionation, and proton-relative biological effectiveness largely remain unaddressed [23].

## Review of evidence

There are numerous reports of the use of PBT for liver OMCs for any primary sources. The most common primaries mentioned in the literature are colo-rectal cancers (CRC), breast cancers (BC), gastric and esophageal cancers and cancers of the biliary tract amongst other cancers. The details of the studies mentioned below have been outlined in Table 1 in a comparative manner.

The Japanese groups were the one of the first to publish large case series and reports regarding the use of PBT in liver secondaries. Kanemoto et al. in an initial report from Fukumitsu et al. group reported five patients treated with a dose of 66 Gray Equivalent (GyE) in 10 fractions or 72.6 GyE in 22 fractions. All the patients tolerated the treatment well, without any grade 3 adverse effects [24]. The highlight from this report was 100% 3-year local control (LC) rates, indicating primarily that liver secondaries from BC show excellent LC rates with PBT and can help in increasing the survival in these hOMCs.

**Table 1:** Comaparative study of 14 publications treating oligometastasis with protons

Author	Study Type (Abstract – Ab/Full paper-FP/Case Report - CR) Nature: Prospective/Retrospective	No of cases/Lesions	Primary Site	Proton Beam Therapy (PBT) doses	Median follow up (months)	LC	PFS	OS	Toxicities	Remarks
Kanemoto et al 2012 (24)	FP/Retrospective	5/7	Breast	66 GyE in 10 fractions or 72.6 GyE in 22 fractions was delivered	33.3	3 yr LC 100%			No grade 3 + toxicities	PBT is a safe and effective treatment for patients with single or multiple, but localized, liver metastases from breast cancer
Fukumitsu et al 2015 (25)	FP/Retrospective	133 cases	Mixed	72.6 GyE/22# & 64 GyE/8# - most commonly used fractionations,	NA	5 yr LC 53%		5 yr OS 24%	NA	The authors claimed that they treated many lesions of more than 5 cm size, with maximum size being 18 cm. Seven of the eight patients with oligometastatic lesion size of more than 10 cm didn’t show local recurrence or Child Pugh score elevation at last reported follow up
Fukumitsu et al 2017 (26)	FP/Retrospective	8 cases	Breast	72.6 GyE/22# & 66 GyE/10#	45	5 yr LC 86%	5 yr PFS 0%	5 yr OS 58%	1 patient suffered a late grade 2 adverse effect (rib fracture),	Protons offer i) Few adverse effects, ii) high local control rate, iii) treatment repeatability and iv) applicable to large tumors
Sufficool et al 2018 (35)	Ab/Retrospective	41/80	Mixed	50Gy / 5 fractions (19%), 30Gy / 5 fractions (16%), and 30Gy / 3 fractions (16%),	14.5	1 year LC was 75%				No RILD, or grade 3 toxicities
Nakajima et al 2019 (39)	Ab/Retrospective	43/53	GI	66 GyE in 10 fractions to a peripherally located tumor and 72.6 GyE in 22 fractions to a centrally located tumor.	20	2 yr LC 70%	NA	2 yr OS 63%	NA	Long systemic therapy – significantly poor OS
Coffman et al 2021 (40)	FP/ Retrospective	46/81	Mixed	3-fraction regimen with dose escalation of 36, 48, and 60 GyE	15	3 yr LC 71.9%		3 yr OS 43.8%	grade 1 and grade 2 toxicity rates were 37% and 6.5% No grade 3 + toxicities	Proton SBRT for the treatment of liver metastases has promising LC rates. Accrual continues for our phase II trial treating liver metastases with Proton SBRT to 60 GyE (Gray equivalent) in 3 fractions
Kim TH et al 2021 (42)	FP/Retrospective	17/18	Breast	60 GyE/10# - for tumour within 2 cm from GI organs 66 GyE/10# - located within 2cm from the hepatic hilum and more than 2cm from GI organs 70-80GyE/10# - located more than 2cm from GI organs & hepatic hilum.	34.2 months		3 yr PFS 19.6%	3 yr OS 71.7%	No grade 3 + toxicities	3 yr FFLP 94.1% PBT can yield promising FFLP and OS rates similar to those resulting from hepatic resection and ablative treatments in Liver metastasis from breast primary
Kim K et al 2022 (45)	FP/Prospective	46/49	Mixed	60 GyE/5# or 70 GyE/10#					No grade ≥3 toxicities	1-year FFLP rate in patients with <3 cm liver metastasis was 87.4%, while that was 74.1% in patients with > 3 cm group (p = 0.087), Hypofractionated PBT with a BED > 100 GyE for liver metastasis is safe and effective
Yamaguchi et al 2020 (46)	FP/Retrospective	7	GI	72.6 GyE/22# was most frequent dosing schedule	41.7	3 year LC 85.7%		3 yr OS 68.6%	no grade 3 to 5 toxicity	PBT appears to be a feasible option for liver oligometastasis from gastric primary
Yamaguchi et al 2023 (47)	FP/Retrospective	41/63 lesions	GI	72.6 GyE/22# was most frequent dosing schedule	27.6	3 year LC 61.6%		3 yr OS 54.9%	no grade 3 to 5 toxicity	PTV must be reduced in tumours near GI tract
Yamaguchi et al 2023 (48)	FP/Retrospective	7/15	GI	72.6 GyE/22# & 64 GyE/8# - most commonly used fractionations, Median fraction size – 3.3 GyE	35	3 yr LC 100%	3 yr PFS 28.6%	3 yr OS 42.9%	No grade > 4	No significant difference between single and multiple metastases
Yamaguchi et al 2023 (52)	FP/Retrospective	24/35	GI	74–76Gy(RBE)/37–38fr 8 72.6–76Gy(RBE)/20–22fr 14 64–66Gy(RBE)/8–10fr - 13 MedianBED10,Gy 96.9(88.8–115.2)	18	3 yr crude LC 94%		3 yr OS 45.3%	No grade ≥3 toxicities	Patients’age (P <0.01),performance status (P =0.017) and tumor size (P = 0.024) were significant OS-related factor
Yamaguchi et al 2024 (53)	FP/Retrospective	14/22	Breast	median dose fractionation used was 6.6 GrayE/#	22.8	2 yr LC 100%	2 yr PFS 62.5%	2 yr OS 33%		Can’t conclude whether PBT is an alternative to systemic therapy for breast primary
Hong TS et al 2017 (56)	FP/Prospective	89	Mixed	30 to 50 Gray equivalent (GyE) in five fractions	30.1 months	3 year LC 71.9%	3 year PFS 9.2%	3 yr OS 20%	no grade 3 to 5 toxicity	Tumor with both mutant KRAS and TP53 were particularly radioresistant, with a one-year LC rate of only 20.0%, compared with 69.2% for all others (p < 0.001).

* **Abbreviations**: Ab – Abstract, FP – Full Paper, LC – Local Control, PFS – Progression Free Survival, OS – Overall Survival, GI – Gastro-intestinal sites, FFLP – Freedom from local progression, PBT - Proton Beam Therapy, SBRT – Stereotactic Body Radiotherapy, BED – Biologically equivalent dose, PTV – Planning Target Volume

Fukumitsu et al. evaluated 140 patients with liver metastasis treated with PBT in a report published in 2015 [25]. The most common primary tumour sites were colon and pancreas. The 5-year OS was 24%, with 2 cases having late adverse effects: rib fracture and cholangitis. For the eighty-five cases with lesions confined to liver, the 5-year overall survival (OS) rate was 28%, whereas, for the remaining patients having extra-hepatic metastasis, the 5-year OS rate was significantly lower at 16% (p = 0.007). In the patient cohort with liver confined oligometastasis, sixty-two cases treated with curative intent had significantly better 5-year OS rate as opposed to the twenty-three cases treated with palliative intent (5-year OS 30 vs 23%; p = 0.016).

The total delivered dose of PBT was 9–77 GyE (with a median of 72.6 GyE). The most frequent dosage used was 72.6 GyE in 22 fractions (used in 72 cases) followed by 66 GyE in ten fractions or 34 lesions. Doses lesser than 66GyE/10 fractions or 72.6 GyE/22 fractions were considered as palliative as per their institutional policy.

One distinguished novel advantage of PBT that this study found, and which was not mentioned in previously published literature on locally ablative therapy or pRT was the size of the curable OMC lesion. The general consensus previously for the oligometastatic lesion size was 5 cm for the feasibility of the locally ablative therapy and SBRT. The authors found that the 5-year OS rate for lesions less than 5 cm in size was 36.8%. The authors claimed that they successfully treated many lesions of more than 5 cm in size, with the maximum size being 18 cm. Seven of the eight patients with an OMC size of more than 10 cm did not show local recurrence or Child Pugh score elevation at the last reported follow-up. The authors also concluded that, for patients with inoperable hepatic metastasis refractory to chemotherapy, local ablative RT is a viable option. This was one of the initial studies highlighting the fact that irradiating hepatic metastasis with the curative intent yields better outcomes compared to when the intent is palliative, largely due to the higher biologically equivalent doses (BED) delivered to the lesions.

Fukumitsu et al. in another report reviewed the patients with hOMC from BC treated with PBT [26]. They reported that five out of eight patients remained alive. The total PBT dose was 66–72.6 GrE. The 3 year-OS was 73%. The authors enumerated the advantages of PBT over other local treatment modalities as follows: high local control rates with fewer adverse effects, possibility of repeating the treatment, and feasibility of applying to large tumours. The authors found the outcomes to be comparable with the outcomes from other local therapy modalities like surgery and TACE. With surgery, the OS rate is 49–75/41–61% at 3/5 years, respectively, with a median of 3.8–5.3 years [27–31]. With TACE, the OS rate was 63–76/13–48% at 1/3 years, respectively [32–34].

Sufficol et al. initially evaluated the efficacy and tolerance for three PBT dose schedules. The doses used were 36 Gy, 48 Gy, and 60 Gy in three fractions. The authors found 60 GrayE in 3 fractions safe in nine cases, and, based on the findings, phase II trial was initiated [35].

A phase 1 study by Kang JI et al. evaluated the maximum tolerated dose of proton SBRT for liver metastasis in nine cases and established the efficacy and safety of 60 GyE in 3-fraction proton beam SBRT in hOMCs [36]. There were no dose limiting toxicities like grade 3 liver or intestinal toxicities observed at all.

The NRG-BR001 consortium [37] used three dose-fraction schedules: 50Gy / 5 fractions (19%), 30Gy / 5 fractions (16%), and 30Gy / 3 fractions (16%), with CRC and BC being the most common sites amongst 80 lesions in 41 cases. With a mean follow-up of 14.5 months, the 1-year LC was 75%, with no RILD detected. Proton SBRT for liver metastases demonstrated a high rate of local tumour control with minimal toxicity. The authors concluded that the integral dose advantage allows patients to have multiple courses of full-dose therapy while still maintaining liver dose constraints. While the initial report from the NRG-BR001 group demonstrated that PBT can be safely executed in OMCs with multiple metastases [38], the above-discussed reports confirmed their safety in treatment with PBT.

Nakajima et al. evaluated image-guided PBT in hOMCs lesions in 53 lesions from 43 cases, with primaries mainly from gastric and colon cancers [39]. With a median follow-up of 20 months, the 2-year OS and LC rates were 63% and 70%, respectively. One of the more interesting observations from this study was that hOMC from the right-sided colon had poorer prognosis compared to those from the left-sided ones, although this has not been validated in further studies available at the time of our research.

Coffman A.R. et al. published a retrospective institutional analysis of eighty-one hOMC from 46 patients treated with PBT [40]. The dose fractionations used were as follows: 27 GyE (27.8%), 30 GyE (16.7%), 36 GyE (19.4%), 48 GyE (19.4%), and 60 GyE (16.7%), and 30 GyE (46.7%) and 50 GyE (40%), for three and five fraction schedules, respectively. With a median follow up of 15 months, the 2-year local control rate was 92.5% without any grade 3 or higher adverse effect. They advocated routine use of deep inspiration breath hold (DIBH), as opposed to 4DCT, for those who can tolerate it, for better outcomes. This report also rebuffed the fact that PBT for liver secondaries gives excellent LC rates.

The authors used 50 Gy/5 fractions for the treatment of hOMC near ribs to reduce the incidence of rib fracture. Additionally, they used 30 Gy/5 fractions to treat hOMC near the stomach and bowel. The authors, however, opine that other alternative options may be used for these cases as they using protons tends to deliver lower Integral doses and hence might result in worse local control rates. The authors found that local control was similar to that of pRT [19,41].

Kim T.H. et al. evaluated the effectiveness of PBT for hOMC from BCs [42]. In the seventeen patients the article discussed, the median tumour size was 2.4 cm. With a median follow-up of 34.2 months, the 3-year freedom from local progression (FFLP) was 94.1%. BCs being a systemic disease, chemotherapy is the preferred modality of choice. Meanwhile, hepatic resection is not an established treatment modality. A recent systemic review of 33 studies, evaluating 965 cases of BC with hepatic metastasis, the evaluated role of hepatic resection and reported median 3-year OS of 52.9% [43]. Although BC treatment is primarily systemic therapy, the role of local treatment cannot be undermined [44]. The authors conclude that PBT offers similar or better FFLP and OS outcomes compared to other ablative treatments and comparable survival outcomes to that of hepatic metastatectomies.

Kim K. et al. treated 46 patients with 49 liver metastases, with the primary sites being CRC, gastrointestinal sites and pancreas/biliary tract [45]. This was a phase II prospective analysis, showing that 1-year FFLP rate in patients with <3 cm liver metastasis was 87.4%, while that was 74.1% in patients with >3 cm group (p = 0.087). None of the patients developed grade ≥3 toxicities. The finding that smaller tumours perform better in terms of LC rates corroborates with those of pRT studies. This report also approves of the fact that >100 GyE of PBT can be safely delivered, without any adverse grade 3 toxicities. The study reported one patient with PBT-induced grade 2 stomach ulceration. While the study involved both intra- and extra-hepatic lesions, patients with intrahepatic metastasis only had superior 1-year OS and PFS. One of the factors which could have possibly confounded the result of this prospective evaluation was the use of systemic agents.

Yamaguchi et al. in 2020 evaluated a similar cohort of seven patients by using 72.6 GraE/22 fractions, with primaries derived from gastric cancers for assessing the efficacy and safety of the proton beam therapy in this cohort [46]. With a median follow-up of 41.7 months, they found that 3-year OS and LC rates were 68.6 and 85.7%, respectively. The outcomes of this small series conclude the important role of PBT in liver secondaries from the gastric origin.

Yamaguchi et al. in another article (published in 2023) discussed the role of protons in hOMCs from CRCs. With a median follow up of 27.6 months, they treated a total of 63 lesions with the most frequent dose being 72.6 GyE/22 fractions [47]. The 3-year LC and OS rates were 61.6 and 54.9%, respectively. Similar to many other studies, they did not find any grade >3 adverse events or any cases of acute liver failure during the early or late phase of radiotherapy. They concluded that, whenever needed, PTV must be reduced because of organ at risk restrictions, especially in tumours near the gastro-intestinal tract. This finding was corroborated by many other studies on PBT.

Yamaguchi et al. analysed esophageal cancers without any extra-hepatic metastasis treated with PBT after primary post-operative therapy [48]. The patient selection criteria were as follows: the primary esophagus carcinoma was resection, metachronous hOMC recurrence without extrahepatic tumours, and no more than three liver metastases. With a median survival of 35.5 months, the most frequent dose was 72.6 GyE/22 fractions for four lesions and 64 GyE /8 fractions for four lesions. The median PFS was 8.7 months. The 2-year OS rate of 57.1% was non-inferior to that of the surgical series by Liu et al. [49], who showed 2-year OS of 21.2%. A few other reports by Huddy and Ichida et al. on local surgical resection showed rates between 20 and 50% [50,51].

Yamaguchi provides another set of data on PBT for hOMC in esophagogastric cancers, in which he reported on 35 hOMCs in six oesophageal and eighteen gastric cancer patients. None of the cases had extra-hepatic or >3 liver lesions. The median survival and 2-year OS rates were 25.3 months and 51.8%, respectively, while the 3-year local recurrence rate was 6%. They concluded that the findings are comparable to surgical outcomes. The most important observation from the study was the absence of grade 3 or higher toxicities for PBT [52].

The most recent publication from the *Japanese Society of Radiation Oncology* on hOMC from breast primary analysed 14 cases with 22 lesions [53]. The median dose fractionation used was 6.6 GyE (range 2-8 GyE) per fraction. The median follow up was 22.8 months. The 2-year LC, OS and PFS rates were 100, 62.5 and 33.3%, respectively. The authors observed that most patients undergoing PBT were either refractory/physically intolerant to chemotherapy, or those who refused to undergo chemotherapy. The authors concluded that it remains unclear as to whether PBT is a better alternative to chemotherapy in cases of hOMCs from breast primary. PBT as, unlike other local treatment options, it can be repeated if required and indicated, as it has been shown from hepatocellular carcinoma [54].

In the times of personalized medicine, the proton therapy cannot be far off from the reality. There have been efforts to inculcate biomarkers to entail a more personalized approach. Therefore, in the future, a more personalized approach similar to other techniques can be expected for protons, although research is very sparse till now.

Ajdari et al. found out that mutation in KRAS oncogene and higher concentration of plasma interleukin-6 at baseline and after fraction 3 were significantly associated with worse local failures after SBRT. Similar studies discuss the need for the incorporation of protons, also in order to better personalize RT for patients [55].

Hong T.S. et al. evaluated the safety and efficacy of risk-adapted stereotactic PBT for liver metastases from solid tumours in 89 cases [56]. The inclusion criteria were patients with limited extrahepatic disease, 800 mL or greater of uninvolved liver, and no cirrhosis or Child-Pugh A. The researchers evaluated the association of common genetic alterations like BRAF, EGFR, HER2, KRAS, NRAS, PIK3CA, and TP53 and tumour characteristics with control rates. Colorectal, pancreatic and gastro-esophageal locations were the most common sites for the primary. The median radiotherapy dose delivered was 40 GyE (range: 30–50 GyE). The median follow up was 30.1 months, and the 3-year local control rate was 61.2%. They observed that the tumour with KRAS mutation had the worst local control rates, while combined KRAS and p53 mutations rendered the tumour highly radioresistant with extremely low 1-year LC rates of 20% (as opposed to 69.2% for the other cohort, p = 0.001). The authors opined on PBT dose escalation to a BED of 100 Gray or higher [57], which has been reported to be a proper dose for pRT for better outcomes and to overcome the radioresistance in the particular cohort with poor local control rates.

RT is a major form of the local therapy in hOMCs, albeit with risk of RILD. Therefore, finding subsets of patients with a higher/lower tolerance of RT might help in identifying a specific subgroup for which dose escalation works better, while sparing radiation damage for more radio-sensitive patients [58,59].

There are case reports suggesting PBT use in oligo-recurrent cancer of esophagus metastasizing to liver. In this particular case report, the authors used omental plombage as a spacer to re-irradiate with protons in a space of 8 months to new liver lesion while using the respiratory gating technique, and they noted an excellent survival of 7 years [60].

Gohongi et al. tried giving concurrent chemotherapy and protons to a hOMC patient with primary gastric cancer. The patient received 66 GyE in thirty fractions along with 5-fluorouracil (250 mg/body per day, as a 24-h intravenous injection for 4 weeks) and low dose cisplatin (10 mg/body on days 1–5 every week for 4 weeks). The patient tolerated the treatment well and was disease-free 2 years post treatment [61].

Muroi et al. reported for the first time the practical use of PBT in hOMC with esophageal primary. They demonstrated high dose delivery by PBT to three liver lesions by using protons, which resulted in a complete response of all three metastases [62].

The Japanese group analysing OMCs published a report in which OMCs from all primaries to three sites were analysed, with data collected from all over the country. This analysis had three arms for comparison, pulmonary, liver and lymph node oligometastasis while comparing the treatment of PBT with that of pRT [63]. In the hOMC arm, one hundred and fifty-one cases with 208 metastatic sites received PBT. The median follow-up was 20.2 months, with the most common sites being CRC, the biliary tract, pancreas, and stomach. The median PBT dose was 109.6 GyE (range 72–394.4 GyE). Although the dose variation in the cohort was high, around 90% of the cases received more than 96 GyE proton therapy doses. The 3-year LC and OS rates were 73.3 and 38.5%, respectively. The risk of grade 3 toxicity was 3.5%, with the most dominant case being dermatitis. No grade 4 toxicities were observed. No statistically significant difference was found in the overall survival, when comparing PBT with pRT in this Japanese nationwide cohort study comparing two modalities of treatments for different oligometastatic sites. However, protons fared better for the colon cancer subset. Another interesting finding from a subset analysis of large (>5 cm) tumours was that PBTs provided durable 3-year local control rates of 84.5%, with an acceptable grade 3 or higher toxicity rate of 2.9%. The specific analysis of hOMC site involved a comparison of six proton-based studies and eleven pRT publications. And, in contrast to protons, very few studies have evaluated pRT for large tumours measuring more than 5 cm, which is a definite advantage of protons. The authors concluded that PBT may be considered as a definitive management choice for >5cm hOMCs.

Colbert et al. in an interesting case series reported on five cases bilateral liver metastases from CRCs treated with PBT. All these cases were not amenable to second stage hepatectomy. Because of the volume of the treatment involved, they labelled the treatment as ‘Hemiliver’ Proton Radiation therapy. Four out of five patients receiving >89.6 GyE achieved partial or complete radiographic response and in-field LC. They concluded that PBT can spare the normal tissue better, and that respiratory gating methods add to the liver sparing [64].

There are no direct randomised studies evaluating pRT with PBT [42]. Meta-analysis for primary liver tumours treated with PBT showed similar OS and FFLP and a lower rate of toxicity compared to Photon-SBRT [65].

### Pooled Survival Analysis

Ten studies involving 393 patients were included in this analysis for overall LC rates. The pooled 3-year LC of 76.8% was covered (95% CI: 65.8–87.8%, I^2^ = 90%). The results are based on studies analysing BC and gastro-intestinal sites. These studies show the best results, while those using a highly mixed subset (which includes other sites along with BC, CRC, stomach) fared worse in terms of the LC rates. The time trend of LC shows high rates in the first two years, with a decline from the 3^rd^ year onwards, with hOMC from BCs maintaining excellent durable local control rates. Drawbacks of the analysis include the inclusion of multiple studies with a small sample size, which can lead to overestimation bias.

In total, eleven studies with 432 cases were evaluated in the analysis of OS data. The pooled data show 3-year OS as 45.2% (95% CI: 35.8–54.6%, I^2^ = 85%). Of these studies, there were only 2 studies reporting hOMCs from BCs, four studies reporting gastro-intestinal sites like oesophagus, stomach and CRC, whereas other evaluations included mixed populations. BC derived hOMCs had better survival rates as compared to other site primaries. The data were highly heterogenous as a lot of studies used mixed patient populations. The high heterogeneity (I^2^ = 85%) was due to the sample size difference (varying from 7 to 133) and site differences across the studies discussed. Another drawback is that small studies from this analysis can cause overestimation bias.

Most of the studies included mixed sites. Subgroup analysis suggests the highest 3-year OS for hOMC from breast primary of 67.6%, (I^2^ = 0%) and the lowest for mixed sites (including CRC, pancreas, stomach, BC and other sites) with 3-year OS of 31.9% (I^2^ = 90%). Again, the mixing of sites along with a high variability raises doubts regarding the results. An individual patient data analysis might be able to answer the conundrum, as to which oligometastasis fares better in a more nuanced way.

The high 3-year LC of 76.8% highlights the proton therapy’s efficacy in controlling hOMC locally, with those derived from BC showing the best outcomes (98.2%). Sites like CRC and BC show relatively better PFS (35–52% at 1–2 years) and OS (33–78% at 1–2 years) than other sites, likely due to disease biology. The contrast with 3-year OS (45.2%) underscores the challenge of systemic disease progression in these cases. It also underlines the role of adding systemic chemotherapy as an important component of oligometastasis treatment.

### Proton versus Photons

Published reports using SBRT to treat hOMC have shown actuarial LC rates ranging from 50–100% [66,67], with higher doses associated with better local control [19]. Multiple published reports have illustrated the distinguished advantage of PBT over pRT for RT of hOMC, the dosimetric advantages of which have enabled the treatment of bigger liver volumes [68–70].

A recently published detailed dosimetric comparison between Cyberknife-based and Proton-based SBRT for liver lesions revealed that protons are best for normal liver sparing for lesions located in the peripheral region or near the chestwall. Protons resulted in significantly lower doses to the spinal cord and chestwall (p<0.05) [71]. Similar findings were echoed by Bonu M.L. et al. in another multi-institutional dosimetric comparison of pRT vs PBT in 213 liver lesions [72].

Another review the role of the proton beam therapy in liver cancers by Choung et al. concluded that protons are a better modality of treatment for liver metastasis and intrahepatic cholangiocarcinoma, compared to X-ray-based stereotactic treatments in terms of the local control, survival outcomes and toxicity rates [70]. A meta-analysis comparing pRT vs. PBT in this particular subset of patients might help in providing a nuanced answer to the question as to which one is a better modality.

The Proton therapy has the advantage of sparing normal liver, especially using gating techniques like DIBH [68,73]. PBT gives excellent target volume coverage and normal tissue sparing with motion management techniques [74]. There are several technical challenges to delivering ultra hypo-fractionated PBT in liver, including type of beams (pencil beam versus scattered beams) and motion management. Motion management is not that well documented for PBT as pRTs [75–78]. PBT had some dosimetric issues initially as compared to pRT in terms of planning and execution, which have already been largely addressed.

Taunk et al. in a case report highlighted the role of complex technicalities involved in PBT delivery planning for two simultaneous liver metastases in a case of postmastectomy oligo-metastatic recurrence [79], including selecting logically proper beam angles and encouraging perpendicular entry to the dominant motion of the patient for better dose-volume coverage. They safely delivered 50 GyE in 5 fractions Proton beam SRT to each lesion, resulting in DFS of 2.5 years post-procedure.

Mondlane et al. in a report on estimating the risk of RILD following the photon or proton therapy concluded that, when using protons, they can spare more healthy liver, lung, kidney, spinal cord and skin as compared to photons dosimetrically. The report was based on ten liver metastasis cases treated with pRTs which were then replanned with PBT. The conclusion was made on the basis of NTCP calculations, and hence the results depend on the proper implementation in clinical practice [80].

### Summarizing the evidence from across different primaries

LC remains persistently high (76–89% at 1–2 years) across BC, CRC, stomach and esophagus. Even the studies having a widely mixed cohort yield decent local control rates. Sites like CRC and BC show relatively better survival outcomes, PFS (35–52% at 1–2 years) and OS (33–78% at 1–2 years) than other sites, likely due to disease biology. Toxicities remain remarkably low with grade 3 plus toxicities ranging from 0–3%, and thus reflecting the proton therapy’s ability to deliver beams with precision. As most of the study types are retrospective, and as none of them being randomised, in terms of comparing PBT to pRTs, especially for hOMCs, the level of evidence remains low.

One of the major drawbacks of this analysis is that most of the studies being analysed were retrospective in nature. The heterogeneity of the analysis was high, and the sample size in some of the studies was very low, further accentuating the final outcome. Many studies used a mixed cohort of a multitude of primaries with hOMCs, thus further confounding the findings and making it difficult to further refine the outcomes.

Some of the advantages which have come to the fore from this analysis and can be utilised as future indications of PBT in hOMCs are: (a) Its use with large-sized tumours of more than 5 cm, with effective sparing of normal liver, (b) repeatability of the procedure, which is not possible in some of the other local therapeutic options for liver oligometastasis, (c) the proton therapy does provide durable and lasting local control in hOMC from any primary site, (d) most importantly, barring a few studies, none of the studies mentioned grade 3 plus toxicities in their reports, which means that its applicability in a wider scale is possible if finances are not a constraint, and lastly (e) as we look towards the future of oncology, it seems inevitable that combining genomic signatures with different modalities including the proton beam therapy might be the best step forward, and that the incorporation of genetic markers and modulation of RT doses can bring the next big revolutionary change in the outcomes of hOMCs.

The analysis concludes that PBT can be safely delivered without compromising the survival outcomes. The outcomes vary for different primary sites. More prospective data are needed to deliver a definitive conclusion.
